# RhoG-Binding Domain of Elmo1 Ameliorates Excessive Process Elongation Induced by Autism Spectrum Disorder-Associated Sema5A

**DOI:** 10.3390/pathophysiology30040040

**Published:** 2023-11-27

**Authors:** Miyu Okabe, Yuki Miyamoto, Yuta Ikoma, Mikito Takahashi, Remina Shirai, Mutsuko Kukimoto-Niino, Mikako Shirouzu, Junji Yamauchi

**Affiliations:** 1 Laboratory of Molecular Neurology, Tokyo University of Pharmacy and Life Sciences, Tokyo 192-0392, Japanmiyamoto-y@ncchd.go.jp (Y.M.); rshirai@toyaku.ac.jp (R.S.); 2 Laboratory of Molecular Pharmacology, National Research Institute for Child Health and Development, Tokyo 157-8535, Japan; 3 Laboratory for Protein Functional and Structural Biology, Center for Biosystems Dynamics Research, RIKEN, Yokohama 230-0045, Japanmikako.shirouzu@riken.jp (M.S.); 4 Diabetic Neuropathy Project, Tokyo Metropolitan Institute of Medical Science,Tokyo 156-8506, Japan

**Keywords:** Sema5A, differentiation, autism spectrum disorder, RhoG, N1E-115

## Abstract

Autism spectrum disorder (ASD) is a neurodevelopmental disorder that includes autism, Asperger’s syndrome, and pervasive developmental disorder. ASD is characterized by poor interpersonal relationships and strong attachment. The correlations between activated or inactivated gene products, which occur as a result of genetic mutations affecting neurons in ASD patients, and ASD symptoms are now of critical concern. Here, for the first time, we describe the process in which that the respective ASD-associated mutations (Arg676-to-Cys [R676C] and Ser951-to-Cys [S951C]) of semaphorin-5A (Sema5A) localize Sema5A proteins themselves around the plasma membrane in the N1E-115 cell line, a model line that can achieve neuronal morphological differentiation. The expression of each mutated construct resulted in the promotion of excessive elongation of neurite-like processes with increased differentiation protein markers; R676C was more effective than S951C. The differentiated phenotypes were very partially neutralized by an antibody, against Plexin-B3 as the specific Sema5A receptor, suggesting that the effects of Sema5A act in an autocrine manner. R676C greatly increased the activation of c-Jun N-terminal kinase (JNK), one of the signaling molecules underlying process elongation. In contrast, the blocking of JNK signaling, by a chemical JNK inhibitor or an inhibitory construct of the interaction of RhoG with Elmo1 as JNK upstream signaling molecules, recovered the excessive process elongation. These results suggest that ASD-associated mutations of Sema5A, acting through the JNK signaling cascade, lead to excessive differentiated phenotypes, and the inhibition of JNK signaling recovers them, revealing possible therapeutic targets for recovering the potential molecular and cellular phenotypes underlying certain ASD symptoms.

## 1. Introduction

Autism is a developmental disorder, presently called autism spectrum disorder (ASD). ASD includes autism, pervasive developmental disorder, and Asperger’s syndrome [1,2,3,4]. The main symptoms of ASD include disturbances in social behavior and interpersonal communication, including repetitive movements and a strong preoccupation with particular objects. In addition, individuals with ASD are often hypersensitive to light and sound stimuli, though in some cases they are insensitive to them. In other words, ASD might be characterized as an integral neuronal disorder of the sensory system [1,2,3,4]. It is believed that ASD is caused by genetic or environmental factors. The majority of ASD cases (50–90%) have familial or sporadic (also called de novo) mutations [5,6,7,8]. The activities of the genetic products may be activated or inactivated by a various of mutations [5,6,7,8]. In either case, it is likely that gene mutations affect the cell morphogenesis of neuronal cells during neurogenesis and neuritogenesis.

In the process of central nervous system (CNS) development, neurons undergo continuous and dynamic cell morphogenesis [9,10]. The morphogenesis involves neurite outgrowth and, in turn, elongation, the navigation of neuronal processes, and synaptogenesis to form neural networks [9,10]. However, the overall molecular mechanisms underlying various neuronal morphological differentiation stages remain incompletely understood [11,12]. In contrast, in neurological diseases, neuronal morphogenesis can be affected, not only at the early stages of development, but also at the later stages and much earlier stages [13,14].

The transmembrane protein semaphorin 5A (Sema5A) serves as an axon guidance cue in response to neuronal growth cone elongation and navigation [15,16,17,18], although Sema5A is one of the bifunctional axon guidance cues that acts as the ligand and sometimes as the receptor [19,20]. Sema5A’s role in autism is well known [21,22,23,24]. Some mutations of the *sema5a* gene are associated with ASD and also epilepsy [25,26]. It is predicted that abnormalities of the *sema5a* gene are directly related to reducing the ability of neurons to form connections between different neuronal cells in some brain regions [27,28]. Herein, we demonstrate that an ASD-associated Arg676-to-Cys (R676C) or Ser951-to-Cys (S951C) mutation of Sema5A excessively promotes process elongation in the N1E-115 cell line, one of the best model lines for neuronal differentiation [29,30], and that the R676C mutation is more effective than the S951C one. We further find that the R676C construct greatly stimulates the phosphorylation levels of c-Jun N-terminal kinase (JNK), which is essential for neuronal cell morphological differentiation [31,32]. Conversely, the inhibition of the JNK signaling pathway, using the upstream molecular interactive domain [33,34], restores excessive process elongation, providing a potential molecular and cellular pathological mechanism underlying Sema5A-related ASD.

## 2. Materials and Methods

### 2.1. Key Antibodies, Chemicals, and Plasmids

The key antibodies and chemicals used in this study are listed in Table 1. The key plasmids used in this study are also listed in Table 1.

### 2.2. Generation of Plasmid-Encoding Mutated Proteins and Other Constructs

The R676C and S951C mutations [24] of Sema5Awere generated from the plasmid-encoding mouse Sema5A (Addgene, Watertown, MA, USA, Cat. No. 72035), using the PrimeStar Mutagenesis Basal kit (Takara Bio, Kyoto, Japan), in accordance with the manufacturer’s instructions. The isolated RhoG-binding domain (RBD, amino acids 1-81) of human Elmo1 was chemically synthesized and fused with pcDNA3.1-N-eGFP (GenScript, Piscataway, NJ, USA). The pET42a-conserved Cdc42 and Rac interactive binding (CRIB) domain of human Pak1 was generated as described [30].

### 2.3. Cell Line Culture, Stable Clone, and Differentiation

A mouse neuronal N1E-115 cell line (JCRB Cell Bank, Osaka, Japan and Japan Health Sciences Foundation, Tokyo, Japan) was cultured on cell culture dishes (Nunc brand of Thermo Fisher Scientific, Waltham, MA, USA) in high-glucose Dulbecco’s modified Eagle medium (DMEM; Nacalai Tesque, Kyoto, Japan) containing 10% heat-inactivated fetal bovine serum (FBS) (Gibco brand of Thermo Fisher Scientific) and penicillin–streptomycin (Nacalai Tesque) in 5% CO_2_ at 37 °C. Cells stably expressing the wild type *sema5a* gene (indicated as WT in the figure) or the gene with the R676C or S951C mutation (indicated as R676C or S951C in the figure) were selected as a single clone in the presence of antibiotic G418 (Nacalai Tesque), in accordance with the manufacturer’s instructions, and cultured without cryopreservation. To induce differentiation, cells were cultured in DMEM and 1% FBS containing penicillin–streptomycin, in 5% CO_2_ at 37 °C for 48 h, unless otherwise indicated. Cells with processes more than one cell body in length were considered to be differentiated process-bearing differentiated cells [30]. Under these conditions, attached cells incorporating trypan blue (Nacalai Tesque) were estimated to be less than 5% in each experiment.

### 2.4. Plasmid Transfection

Cells were transfected with the plasmids using the ScreenFect A transfection kit (Fujifilm, Tokyo, Japan), in accordance with the manufacturer’s instructions. The medium was replaced 4 h after transfection and was generally used for 48 h after transfection for cell biological and biochemical experiments, unless otherwise indicated. Under these conditions, attached cells incorporating trypan blue were estimated to be less than 5% in each experiment.

### 2.5. Preparation of Cell Extracts or Lysates, Fractionation, and Denatured Polyacrylamide Electrophoresis and Immunoblotting

Cell extracts were prepared using a Potter-Elvehjem homogenizer in extraction buffer (50 mM HEPES-NaOH, pH 7.5, 150 mM NaCl, 5 mM MgCl_2_, 1 mM dithiothreitol, 1 mM phenylmethanesulfonyl fluoride, 1 microgram/mL leupeptin, 1 mM EDTA, 1 mM Na_3_VO_4_, 10 mM NaF, and 0.25% sodium cholate). The extracts were subjected to ultracentrifugation. The supernatants and precipitates were collected separately and precipitates were gently lysed in lysis buffer (50 mM HEPES-NaOH, pH 7.5, 150 mM NaCl, 5 mM MgCl_2_, 1 mM dithiothreitol, 1 mM phenylmethanesulfonyl fluoride, 1 microgram/mL leupeptin, 1 mM EDTA, 1 mM Na_3_VO_4_, 10 mM NaF, and 0.5% NP-40). Equal amounts (20 micrograms per sample) of the supernatants and the precipitates were used for the following experiments. For other experiments, cells were collected and lysed in lysis buffer. The supernatants (20 micrograms per sample) or samples were denatured in sample buffers (Fujifilm). The samples were separated on a sodium dodecyl sulfate–polyacrylamide (SDS-PAGE) gel (Nacalai Tesque). The electrophoretically separated proteins or the resultant precipitates were transferred to a polyvinylidene fluoride (PVDF) membrane (Fujifilm), blocked with Blocking One (Nacalai Tesque), and immunoblotted using primary antibodies, followed by peroxidase enzyme-conjugated secondary antibodies. Peroxidase-reactive bands were captured using an image scanner (Canon, Tokyo, Japan) and scanned using CanoScan software ver. 1.1 (Canon). The blots shown in the figures are representative of three blots. We performed some sets of experiments for immunoblotting studies and quantified other immunoreactive bands with one control’s immunoreactive band as 100%, using Image J software (ver. Java 8, https://imagej.nih.gov/ accessed on 10 April 2023).

### 2.6. Affinity Precipitation Assay to Monitor Guanosine Triphosphate (GTP)-Bound Rac1 Protein

First, *Escherichia coli*-generated, recombinant glutathione-s-transferase (GST)-tagged CRIB domain (20 mg per sample) was mixed with glutathione resin (Thermo Fisher Scientific) and washed with lysis buffer [30]. The CRIB domain specifically binds to active, GTP-bound Rac1. Then, the supernatants of the cells lysed with lysis buffer (800 micrograms per sample) were mixed with GST-CRIB-absorbed glutathione resin, collected by centrifugation, and washed with lysis buffer. The washed resins were denatured in sample buffers, applied to SDS-PAGE gels, and blotted to PVDF membranes in order to detect active, GTP-bound Rac1 by immunoblotting.

### 2.7. Fluorescent Images

Cells on coverslips were fixed with 4% paraformaldehyde (Nacalai Tesque) or 100% cold methanol (Nacalai Tesque) and blocked with Blocking One. Slides were incubated with primary antibodies preloaded with fluorescent dye-conjugated secondary antibodies. The coverslips were mounted using the Vectashield kit (Vector Laboratories, Burlingame, CA, USA). The fluorescent images were collected and merged with microscope systems FV1200 or FV3000 equipped with a laser-scanning Fluoview apparatus and software (both from Olympus, Tokyo, Japan). The images in the figures are the representative of three images and were analyzed using Image J software.

### 2.8. Statistical Analyses

Values are shown as means ± standard deviations (SD) of separate experiments. Intergroup comparisons were made using the unpaired Student’s *t*-test in Excel (ver. 2021, Microsoft, Redmond, WA, USA). A one-way analysis of variance (ANOVA) was followed by a Tukey’s multiple comparison test using Graph Pad Prism (ver. 5, GraphPad Software, San Diego, CA, USA), if multiple comparison was necessary. Differences were considered statistically significant when *p* < 0.05.

### 2.9. Ethics Statement

Techniques using genetically modified cells and related techniques were performed in accordance with a protocol approved by the Tokyo University of Pharmacy and Life Sciences Gene and Animal Care Committee (Approval Nos. LS28-20 and LSR3-011).

## 3. Results

### 3.1. Mutated Sema5A Preferentially Localizes around the Plasma Membrane

**Figure 1:**
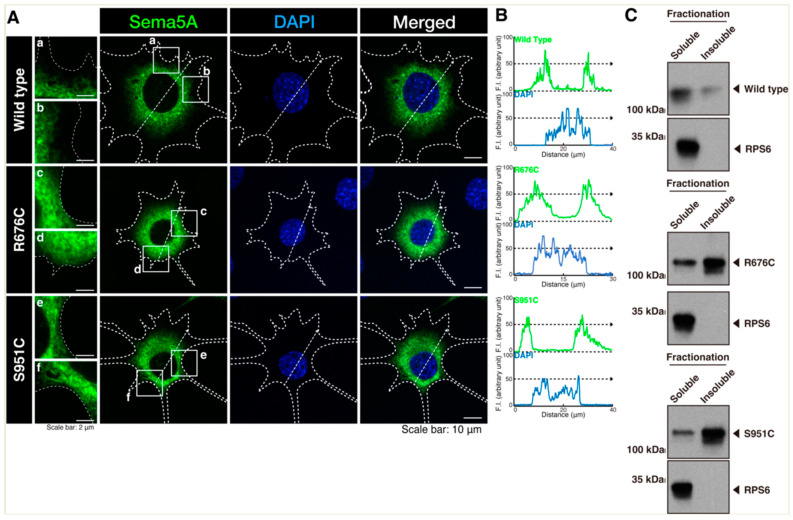
Mutated but not wild type Sema5A protein is preferentially localized around the plasma membrane. (**A**,**B**) N1E-115 cells (surrounded by white dotted lines) were transfected with the plasmid-encoding His-tagged wild type Sema5A or Sema5A with the R676C or Ser951C mutation. Transfected cells were stained with an anti-His antibody (green) and DAPI (blue) to detect nuclear position. Images in small squares (a–f) are enlarged in the left panels (a–f). Graphs showing fluorescence intensities (F.I.; arbitrary units) along the dotted lines in the direction of the arrows are depicted in the right panels. (**C**) Soluble or insoluble fractions of the transfected cell extracts were fractionated by centrifugation. Equal amounts of fractionated proteins were immunoblotted with an antibody against His-tagging. An anti-ribosomal protein S6 (RPS6) antibody was used for detecting soluble proteins as the positive controls.

**Figure 2:**
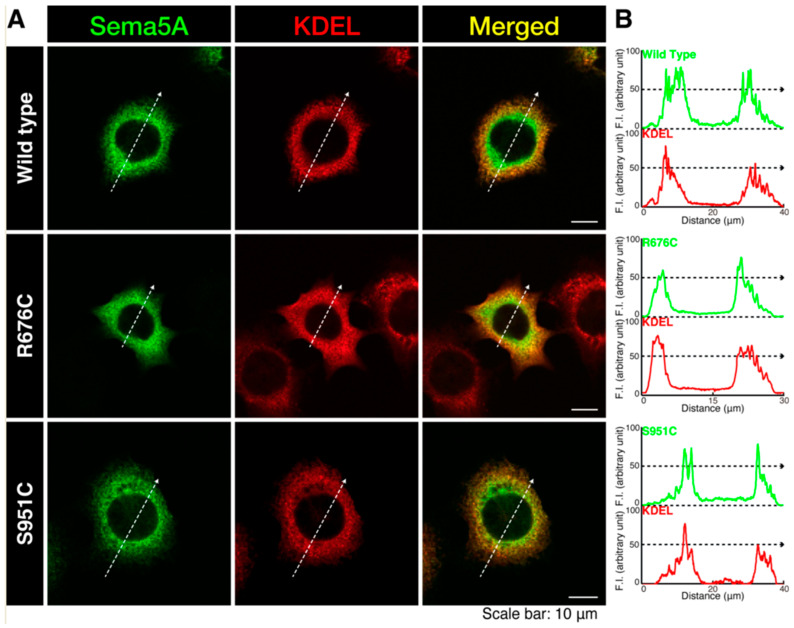
Wild type and mutated Sema5A proteins are partially localized in the endoplasmic reticulum. (**A**,**B**) Cells (surrounded by white dotted lines) were transfected with the plasmid-encoding His-tagged wild type Sema5A or Sema5A with the R676C or Ser951C mutation. Transfected cells were stained with an antibody against His antigen (green) and KDEL antigen (red) to detect ER position. Graphs showing fluorescence intensities (F.I.; arbitrary units) along the dotted lines in the direction of the arrows are depicted in the right panels.

**Figure 3:**
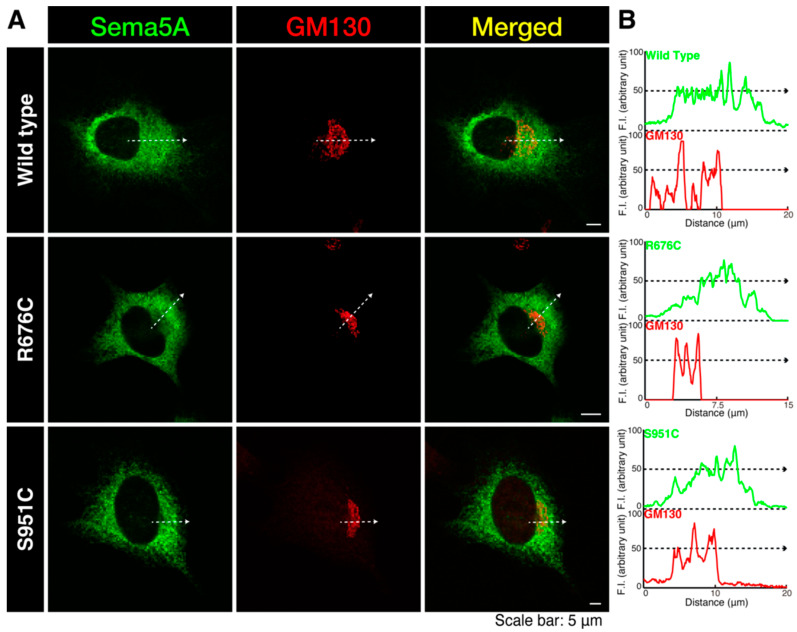
Wild type and mutated Sema5A proteins are partially localized in the Golgi body. (**A**,**B**) Cells (surrounded by white dotted lines) were transfected with the plasmid-encoding His-tagged wild type Sema5A or Sema5A with the R676C or Ser951C mutation. Transfected cells were stained with an antibody against His antigen (green) and GM130 antigen (red) to detect Golgi body position. Graphs showing fluorescence intensities (F.I.; arbitrary units) along the dotted lines in the direction of the arrows are depicted in the right panels.

To investigate whether an ASD-associated mutation of Sema5A leads to change in the cellular localization of Sema5A, we transfected the plasmid-encoding wild type Sema5A or a Sema5A harboring the R676C or S951C mutation into N1E-115 cells. Wild type Sema5A was localized in the cytoplasmic region, whereas Sema5A harboring the R676C or S951C mutation was localized throughout the cytoplasmic region and was also present around the plasma membrane (Figure 1A,B). These results were consistent with the following biochemical experiments. Wild type Sema5A was preferentially present in soluble fraction, whereas mutated Sema5A (R676C or S951C) was preferentially present in insoluble fraction. It is likely that mutated proteins are preferentially localized in the plasma membrane. We also stained wild type Sema5A or Sema5A harboring the R676C or S951C mutation with an antibody (anti-Lys-Asp-Glu-Leu [KDEL] antigen) specific to the endoplasmic reticulum (ER). Wild type Sema5A or Sema5A harboring the R676C or S951C mutation exhibited partial localization with an ER marker (Figure 2A,B). We further stained wild type Sema5A or Sema5A harboring the R676C or S951C mutation with an antibody (a 130 kDa Golgi body membrane surface protein [GM130] antigen) specific to the Golgi body. All Sema5A constructs exhibited partial localization with a Golgi body marker (Figure 3A,B). Since Sema5A is a transmembrane protein [15,16,17,18,19,20], the wild type or mutated protein is present on the secretary pathway; the mutated protein can be preferentially transported around the plasma membrane.

### 3.2. Mutated Sema5A Excessively Leads to Morphological Differentiation

**Figure 4:**
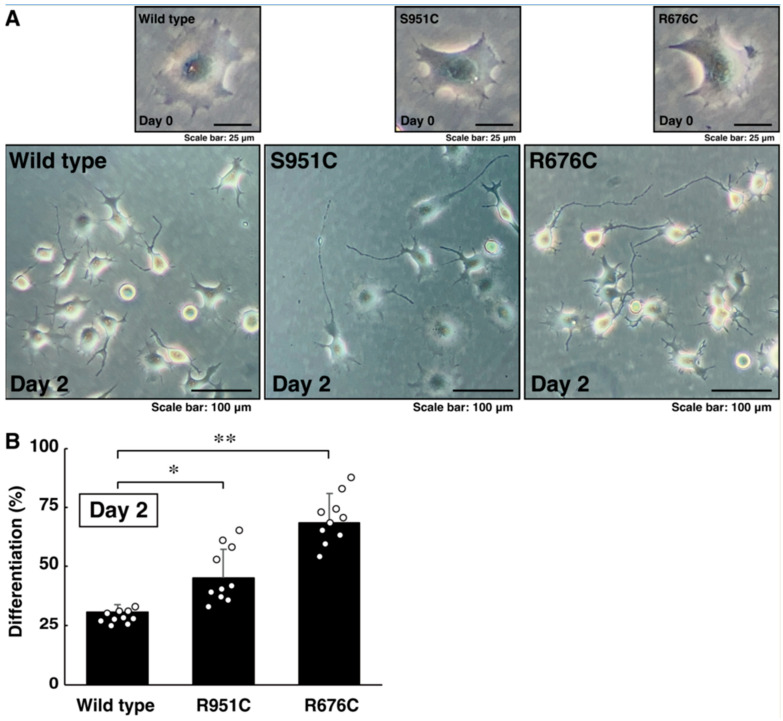
Mutated Sama5A triggers excessive neurite-like process elongation. (**A**,**B**) Cells harboring wild type Sema5A or Sema5A with the R676C or Ser951C mutation were allowed to differentiate for 0 or 48 h. Cells with processes with a body length of more than one cell were counted as cells with neurite-like process elongation and are statistically depicted in the graph (* *p* < 0.05, ** *p* < 0.01; n = 10 fields).

**Figure 5:**
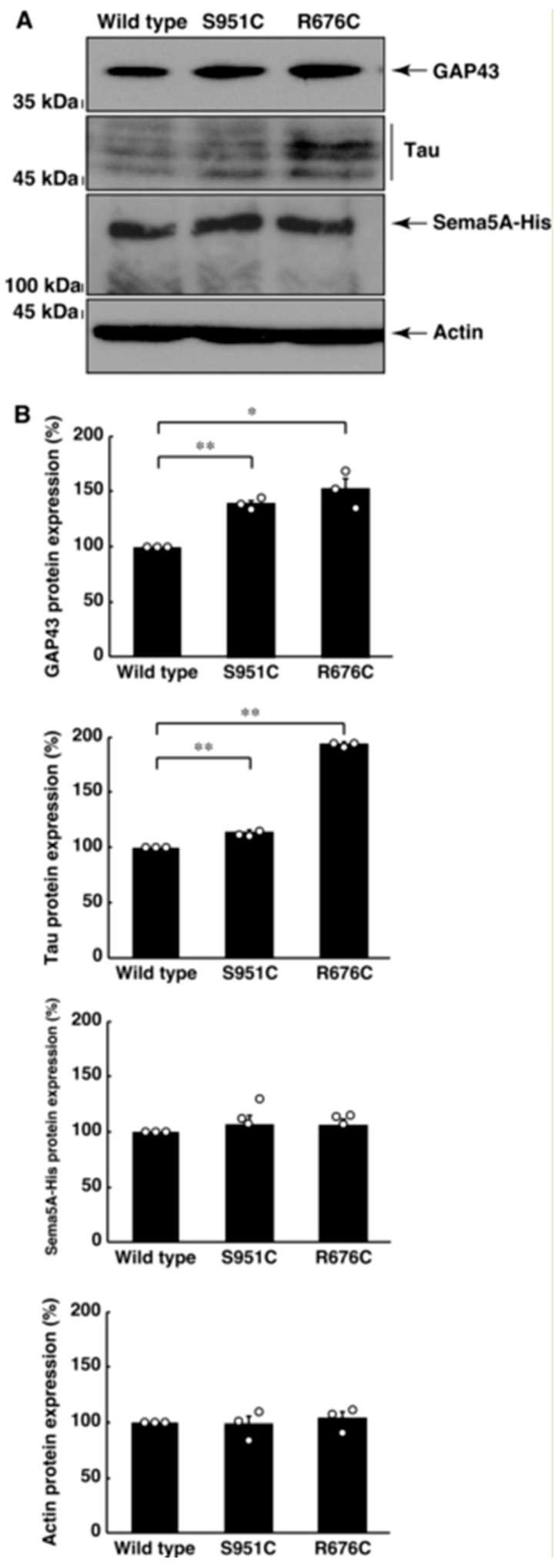
Mutated Sama5A excessively increases neuronal differentiation marker expression. (**A**,**B**) The lysates of cells following the induction of differentiation were immunoblotted with an antibody against a neuron differentiation marker protein (GAP43 or Tau), His-tag, or the internal marker actin protein. Their immunoreactive band intensities are statistically depicted (* *p* < 0.05, ** *p* < 0.01; n = 3 blots). An anti-Tau antibody recognizes approximately 50 kDa of many Tau protein variants. Other immunoreactive bands can be non-specific ones.

Since mutated Sema5A exhibited preferred localization around the plasma membrane, mutated Sema5A (R676C or S951C) may be more effective than wild type Sema5A for neuronal cell morphological differentiation. We allowed cells expressing the wild type or mutated protein (R676C or S951C) to differentiate for 2 days. Cells expressing the wild type protein achieved approximately 25% differentiation phenotypes, such as neurite-like process elongation (Figure 4A,B). On the other hand, cells expressing R676C or S951C protein achieved approximately 50% or 70% differentiation phenotypes, respectively (Figure 4A,B). Since the effect was, at least in part, inhibited by the pretreatment with the respective antibodies against Plexin-B3, Plexin-A2, and Plexin-A3 (Figure S1), this implies that all Sema5A receptor candidates reported previously [35,36,37,38] likely contribute to responses to Sema5A. In addition, in cells expressing the wild type, R676C, or S951C proteins, neuronal differentiation markers’ growth-associated protein 43 (GAP43) and Tau were increased, following the induction of differentiation (Figure 5A,B). In particular, cells expressing the S951C protein significantly upregulated the expression levels of differentiation markers. The expression levels of the control, actin protein, were comparable in all cells (Figure 5A,B). These results indicate that Sema5A harboring the R676C or S951C mutation has the ability to enhance differentiation. Additionally, Sema5A harboring the R676C mutation is more effective than Sema5A harboring the S951C mutation.

**Figure 6:**
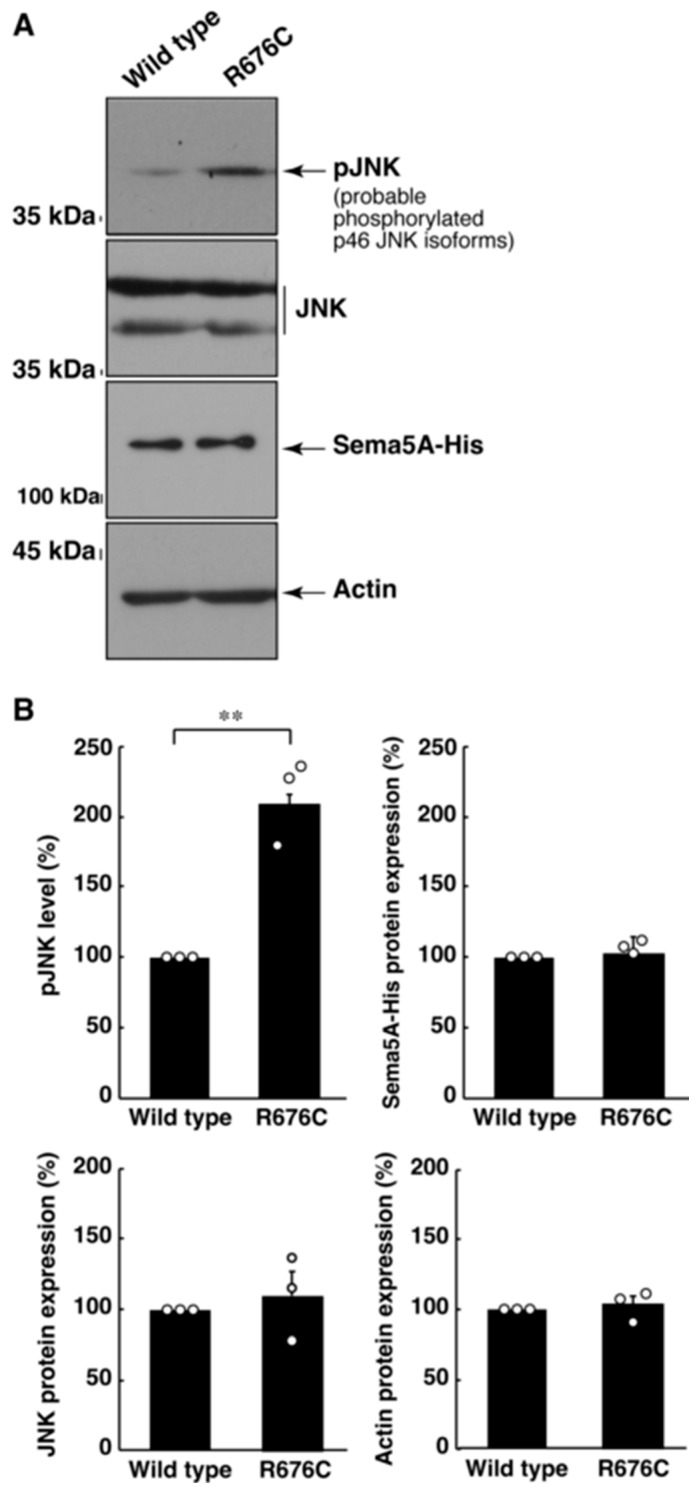
Mutated Sama5A excessively increases JNK phosphorylation. (**A**,**B**) The lysates of cells, following the induction of differentiation, were immunoblotted with an antibody against active phosphorylated JNK (pJNK), JNK, His-tag, or actin protein. Their immunoreactive band intensities are statistically depicted (** *p* < 0.01; n = 3 blots). In the phosphorylated JNK blot, this antibody detected only probable 46 kDa isoforms of JNK proteins.

We asked whether Sema5A harboring the S951C mutation could enhance the activation of JNK. JNK is well known to be one of the major signaling molecules that undergoes neuronal morphological differentiation [31,32]. Sema5A harboring the R676C mutation had the ability to enhance JNK phosphorylation, which was critical for its activation [31,32], compared to the wild type Sema5A (Figure 6A,B). We suggest that the R676C mutation of Sema5A is involved in accelerating differentiation phenotypes.

### 3.3. RhoG-Binding Domain of Elmo1 Can Recover the Excessive Differentiation Phenotypes

**Figure 7:**
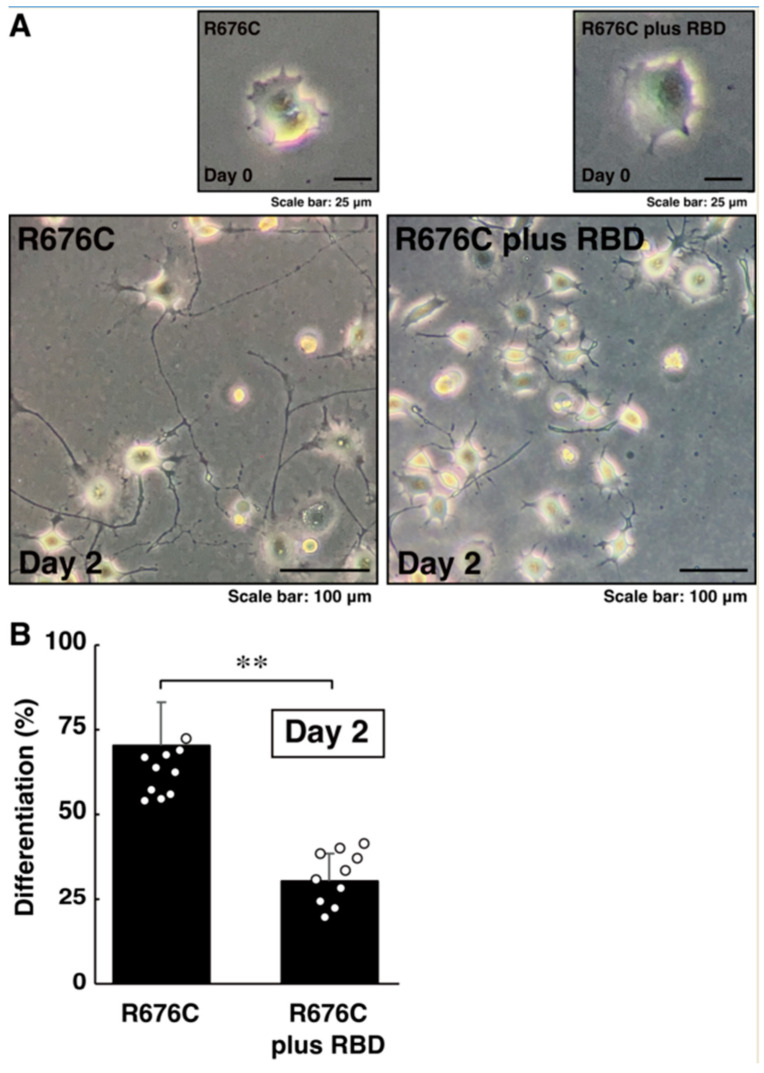
RBD recovers excessive process elongation induced by Sama5A harboring the R676C mutation. (**A**,**B**) Cells harboring Sema5A with the R676C mutation were transfected with the plasmid-encoding RBD and allowed to differentiate for 0 or 48 h. Differentiated cells are statistically depicted in the graph (** *p* < 0.01; n = 10 fields).

**Figure 8:**
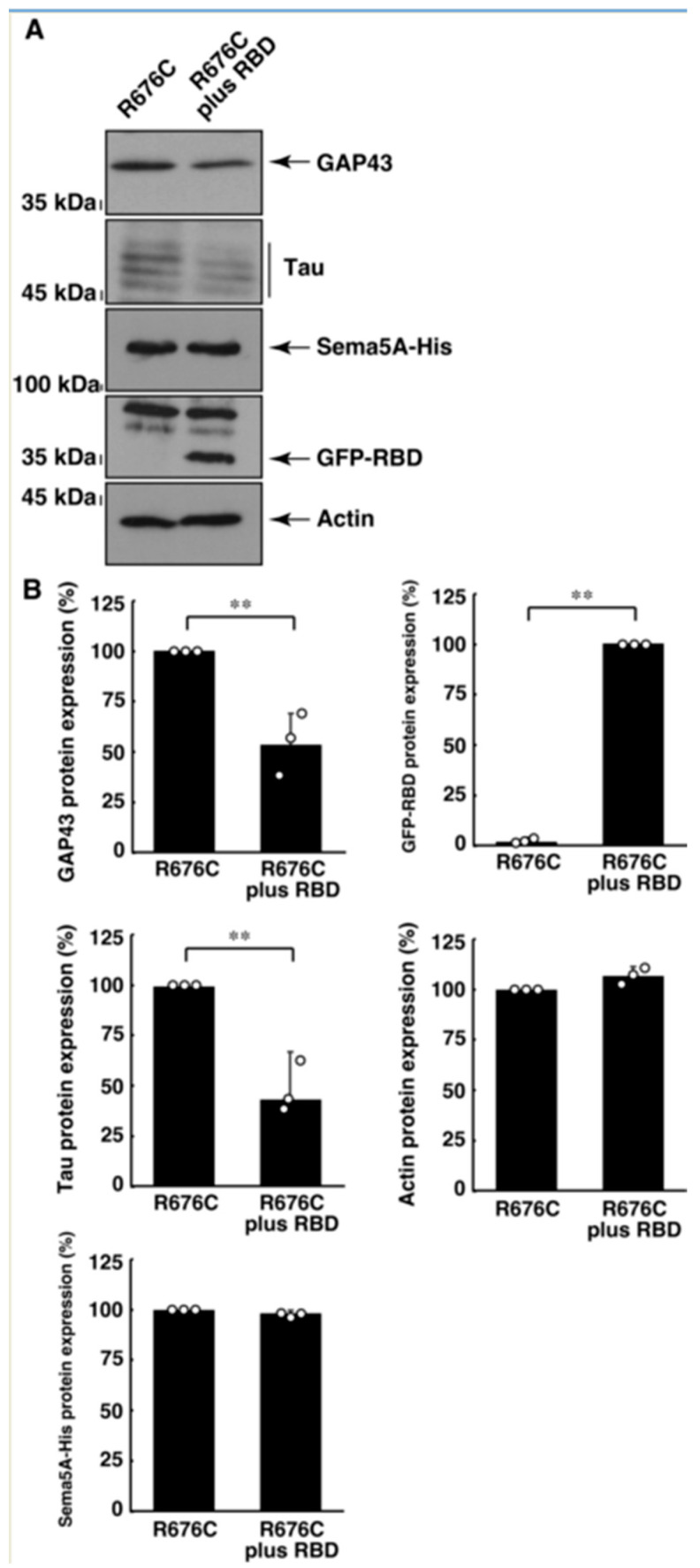
RBD recovers excessive neuronal differentiation marker expression induced by Sama5A harboring the R676C mutation. (**A**,**B**) The lysates of cells following the induction of differentiation were immunoblotted with an antibody against GAP43, Tau, His-tag, GFP-tag, or actin protein. Their immunoreactive band intensities are statistically depicted (** *p* < 0.01; n = 3 blots). An anti-Tau antibody recognizes approximately 50 kDa of many Tau protein variants. Other immunoreactive bands can be non-specific ones.

**Figure 9:**
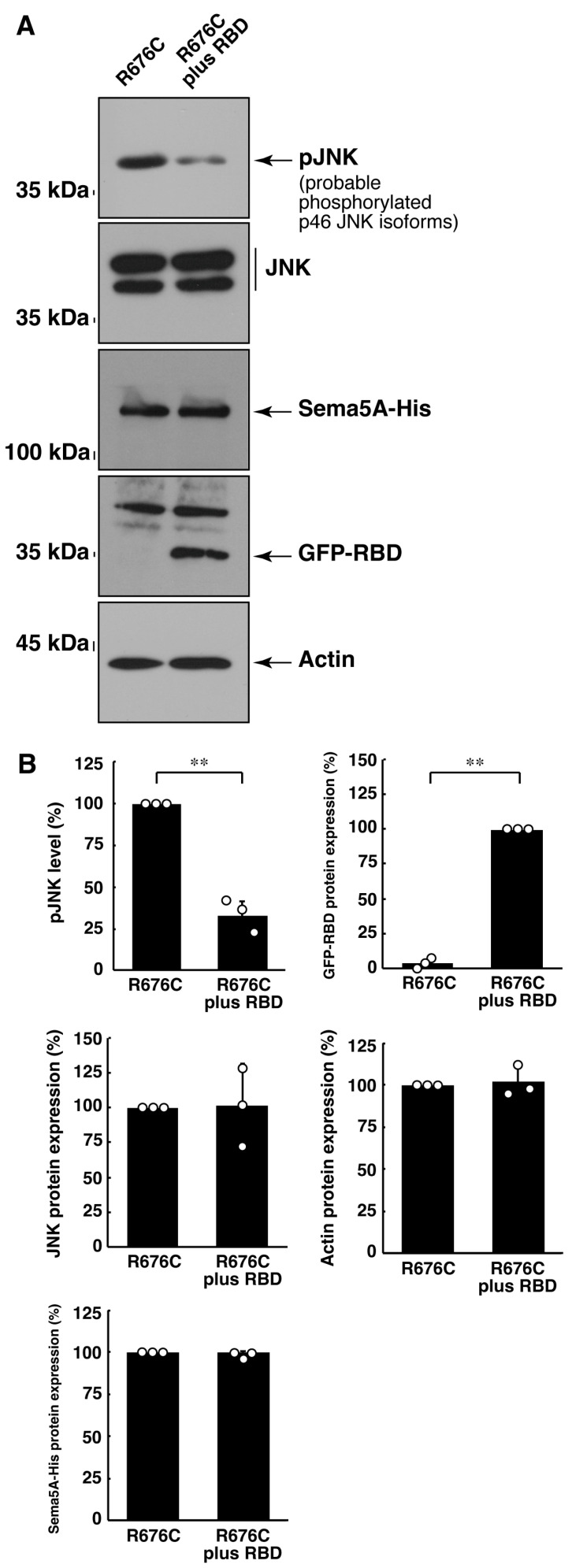
RBD recovers excessive JNK phosphorylation induced by Sama5A harboring the R676C mutation. (**A**,**B**) The lysates of cells, following the induction of differentiation, were immunoblotted with an antibody against phosphorylated JNK, JNK, His-tag, GFP-tag, or actin protein. Their immunoreactive band intensities are statistically depicted (** *p* < 0.01; n = 3 blots).

Next, in order to investigate whether some inhibitors potentially recover excessive differentiation phenotypes, we focused on the R676C mutation of Sema5A, which has strong effects. We first tried to examine the effects of curcumin as a JNK inhibitor on neuronal cell morphological differentiation. Curcumin is a yellow polyphenol compound, found in plants such as turmeric, which has multiple effects on mammalian cells. One of its known effects is the inhibition the JNK singling pathway [39]. Since this effect is mild, it is a suitable choice to investigate the involvement of intracellular JNK-mediated signaling. For cells expressing the R676C mutant, treatment with curcumin recovered differentiation to the differentiation level of cells expressing the wild type (Figure S2). Similar effects were observed in the expression levels of markers, although changes in GAP43 were small but significant (Figure S3). Thus, we transfected the plasmid-encoding construct (RBD of Elmo1) to block RhoG, which is present in the most upstream JNK signaling pathway [33,34], into cells expressing the R676C mutant. The results revealed that RBD recovered the levels of differentiation phenotype (Figure 7A,B) and marker expression (Figure 8A,B) seen in cells expressing the wild type. Similarly, the phosphorylation levels of JNK were recovered (Figure 9A,B). We further confirmed the activities of Rac1 as the effector acting downstream of RhoG and Elmo1. In an affinity precipitation assay in cells expressing the R676C mutant, active GTP-binding Rac1 levels were decreased to those seen in cells expressing the wild type, following the transfection with RBD (Figure S4).

Taken together with studies that used inhibitors of upstream signaling molecules of JNK signaling and JNK itself, these results indicate that the inhibition of signaling through JNK has the ability to recover excessive differentiation phenotypes by the ASD-associated R676C mutation of Sema5A, at least at the in vitro level.

## 4. Discussion

Using cDNA microarray technology, some transcripts, including the *sema5a* gene, have been identified to be downregulated in autism patients [21]. Deletion of the chromosome 5′s short arm region has been reported to be associated with phenotypic features including microcephaly, intellectual disability, and a cat-like cry in infancy (called cri du chat syndrome) [22]. The deleted region includes the *sema5a* gene and some other genes. The genomic region also contains the *sema5a* gene that is associated with causing infantile epilepsy [25,26]. It has also been found that, in autistic patients [23,24], the Arg-676 and Ser-951 positioned between the extracellular domain and the intracellular region in Sema5a are replaced by the Cys residue, which participates in forming a potential disulfide bond in the extracellular region and in providing a reducing group in the intracellular region. The structure and function of Sema5A are modified by their amino acid replacements in autism. Genetic mutations possibly affecting neurons in ASD patients may cause imbalances between excitatory and inhibitory neurons, between neurons and glia, and between neurons and immune cells in the brain [1,2,3,4]. In any case, the abnormal development of the neurons themselves seems likely to participate in the formation of ASD as a neurodevelopmental disorder. In this study, using the N1E-115 cell line as the model, we identified that Sema5A harboring the R676C or S951C mutation exhibits a preferred localization around the plasma membrane. The aberrant properties may allow mutated proteins to promote neuronal morphological differentiation, typically showing neurite-like process elongation. The promoting phenotypes are supported with increased neuronal differentiation marker expression, implying that the excessive elongation of processes is related to the changes in the biochemical properties of Sema5A, induced by ASD-associated R676C or S951C mutation.

Symptoms of ASD are typically noticed during the second year of life (around 12–24 months) but can be noticed earlier or later [1,2,3,4]. Analysis using Sema5A knockout mice illustrates that the function of Sema5A is to inhibit synaptogenesis in hippocampal dentate granule cells, but only in the early postnatal stage [27,28]. The knockout of Sema5A leads to excessive synaptogenesis [27,28]. Synapse formation includes continuous and dynamic localized cell morphological changes. These changes are likely to be consistent with the biological and biochemical properties of Sema5A, induced by ASD-associated R676C or S951C mutation. The suitable inhibitory effects of Sema5A on synapse formation may lead to the retainment of axon elongation, and the possible related morphological changes, in the early periods of brain development. This phenomenon may result in the formation of sufficient networks between synapses in the later developmental periods, although it remains to be determined how much Sema5A knockout mice reflect early onset autism and the developmental abnormality in humans [27,28]. Further analyses using various types of Sema5A genetically modified mice will allow us to determine whether knockout or transgenic Sema5A mice reflect early or later onset autism, or both types. Alternatively, for example, knockin mice, harboring the R676C or S951C mutation of Sema5A, may actually reflect some types of autism in humans.

Polymorphism in the promoter region of Sema5A has been found in some ASD patients. Despite the unknown role of polymorphism on the transcriptional and possible translational levels of Sema5A, it is believed that the transcriptional regulation of Sema5A is associated with the onset of ASD [40]. In one study, the forced or regulatory expression of the anti-apoptotic Bcl-2 or c-Myb proto-oncogene product’s transcription factor participates, transcriptionally and translationally, in sustaining increased levels of Sema5A and contributes to the progression of melanoma, which is derived from cells in the skin called melanocytes, from neural crest cells [41]. Therefore, fine-tuned control of the promoter region of Sema5A, and thus the amount of wild type or possible mutated Sema5A protein, is predicted to play an important role in maintaining cellular homeostasis, differing depending on cell types and/or on healthy and pathologically developmental states.

We found that the neutralizing antibody for the Plexin-B3 receptor, specific to Sema5A [35], at least in part inhibits excessive processes elongation that is induced by preferential plasma membrane-residential R676C or S951C mutations of Sema5A. It is possible that the plasma membrane-residential Sema5A mutant protein binds to Plexin-B3 in an autocrine or paracrine manner, resulting in excessive process elongation in cells. Similarly, a neutralizing antibody for Plexin-A2 or Plexin-A3 [37], as a potential Sema5A receptor, only partially inhibits excessive process elongation. It is thus clear that Sema5A mutant protein promotes process elongation in an autocrine or paracrine manner, although Sema5A may bind to different receptors of the Plexin family than those previously expected.

Among Plexin-B3, Plexin-A2, and Plexin-A3, the relationship between Plexin-A2, Plexin-A3, and ASD is known [42,43]. Whole exome sequencing analysis, in an ASD patient with complex neurobehavioral phenotypes, who also had epilepsy and another attention disorder, clarified that the Arg-205-to-Gln (R205Q) and Arg-1653-to-Gln (R1635Q) mutations of Plexin-A2, as well as the Leu-487-to-Pro (L487P) mutation of a functionally unidentified protein leucine-rich repeat containing 40 (LRRC40), were critically associated with these symptoms, including ASD. In contrast, genomic sequencing analyses in ASD patients with neurodevelopmental disorders identified 26 novel candidate genes, including Plexin-A3, which has now been found to be associated with intellectual disability syndrome [44]. It is conceivable that these mutations of Plexin family receptors are responsible for excessive process elongation in cell lines and axon elongation in primary cells. Apart from the autocrine or paracrine binding manners in Sema5A, some abnormities in Sema5A and its cognate Plexin receptor interaction can trigger an abnormal neuronal network, possibly leading to abnormalities of brain function.

It is known that, in unique cases, Plexin-B3 interacts in a homophilic manner, to be activated in an epithelial-like COS-7 cell line [45]. That is to say, Plexin B3 is activated without binding to Sema5A and triggers downstream signaling. Although this homophilic interaction results in the promotion of neurite outgrowth in primary mouse cerebellar neurons, the detailed analyses of intracellular signaling induced by homophilic and heterophilic interactions has been studied in other types of cells [46]. For example, Plexin-B3, acting through a variety of small GTPases belonging to Ras and Rho families, is involved in the regulation of cell motility and metastasis in glioma [47]. Furthermore, Plexin-B3, acting together with the interaction with the Met proto-oncogene product’s receptor tyrosine kinase, promotes cancer stem cell motility and metastasis [48,49,50]. This signaling is mediated by the non-receptor tyrosine kinase c-Src and focal adhesion kinase (FAK). It is likely that Plexin-B3 itself governs the major intracellular signaling molecules, coupling tyrosine kinases to small GTPases in cells.

Signals that couple receptor or non-receptor tyrosine kinases to small GTPases often contain JNK signals [31,32]. In addition, JNK is one of the central signaling molecules controlling axon elongation. For example, JNK, acting through Rac1 and Cdc42, promotes process elongation in N1E-115 cells [30]. In this pathway, genetically conserved Dock family molecules primarily act upstream of Rac1 and/or Cdc42 and JNK [33]. Dock180 (also called Dock1), Dock2, and Dock5 belong to the Dock-A subfamily and play roles in activating Rac1 and its downstream JNK [33]. In this study, we asked whether blocking these signals could lead to recover excessive process elongation. Because there exist some signaling molecules upstream of Rac1 and JNK, we have utilized the isolated RhoG-binding domain of Elmo1 (tentatively called RBD in this study) to block all Dock-A subfamily molecules [33,34]. Elmo1 is an adaptor protein between upstream RhoG and downstream Dock-A subfamily molecules. We found that RBD has the ability to recover excessive process elongation. Similar results are obtained in the case of a JNK inhibitor. RBD may be classified into lists of unique types of protein–protein interaction inhibitors (PPIs) for excessive process elongation.

Herein, we show that Sema5A protein harboring ASD-associated R676C or S951C mutation, but not wild type Sema5A, is preferentially localized around the plasma membrane. The mutation has the ability to undergo excessive neuronal cell morphological differentiation with an increased differentiation marker expression. The inhibition of JNK signaling by chemical inhibitors or RhoG-binding domains recovers excessive differentiation, illustrating that molecules belonging to signaling through JNK could be a potential therapeutic target for related disorders. Additional studies allow us to promote our understanding, not only of the mechanism by which the ASD-associated Sema5A mutant protein promotes neuronal differentiation, but also of how Sema5A itself promotes differentiation in vitro and in vivo. The series of studies help elucidate how wild type or mutant Sema5A modulates signal transduction pathways from the RhoG/Elmo1/Dock-A subfamily to JNK and unidentified transcription factors. Such studies may lead to the development of possible drug target-specific medicine for ASD and related diseases.

## Figures and Tables

**Table 1:** Key materials used in this study.

Reagents or Materials	Companies or Sources	Cat. Nos.	Lot. Nos.	Concentrations Used
Key Antibodies				
Ant-growth-associated protein 43 (GAP43)	Santa Cruz Biotechnology (Santa Cruz, CA, USA)	sc-17790	J0920	Immunoblotting (IB), 1:5000
Anti-Tau	Santa Cruz Biotechnology	sc-21796	H2721	IB, 1:500
Anti-Tau	Santa Cruz Biotechnology	sc-21796	G1222	IB, 1:500
Anti-actin (also called pan-β type actin)	MBL (Tokyo, Japan)	M177-3	007	IB, 1:5000
Anti-c-Jun N-terminal kinase (JNK, pan-JNK [p54 isoforms])	Santa Cruz Biotechnology	sc-7345	J2521	IB, 1:250
Anti-phospho-c-Jun N-terminal kinase (pJNK, pan-pJNK [phosphorylated p54 isoforms])	Santa Cruz Biotechnology	sc-6254	C1722	IB, 1:500
anti-Rac1	Proteintech Japan (Tokyo, Japan)	66122-1-Ig	10011346	IB, 1:500
anti-His	MBL	D291-3	002	IB, 1:500
Anti-Lys-Asp-Glu-Leu (KDEL)	MBL	M181-3	004	Immunofluorescence (IF), 1:200
Anti-130 kDa Golgi membrane protein (GM130)	BD Biosciences (Franklin Lakes, NJ, USA)	610823	8352796	IF, 1:200
Anti-cathepsin D	Abcam (Cambridge, UK)	ab75852	GR260148-33	IF, 1:200
Ribosomal protein S6 (RPS6)	Santa Cruz Biotechnology	sc-74459	D2921	IB, 1:500
Anti-Plexin-A2	Santa Cruz Biotechnology	sc-39393	F0719	Neutralization, 1 microgram per 1 ml of medium
Anti-Plexin-A3	Santa Cruz Biotechnology	sc-37466	C1923	Neutralization, 1 microgram per 1 ml of medium
Anti-Plexin-B3	Atlas Antibodies (Bromma, Sweden)	HPA048046	23445	Neutralization, 1 microgram per 1 ml of medium
IgG (Mouse control IgG)	Cosmo Bio (Tokyo, Japan)	65124-1-lg	U0000635	Neutralization, 1 microgram per 1 ml of medium
Anti-IgG (H+L chain) (Rabbit) pAb-HRP	MBL	458	353	IB, 1:5000
Anti-IgG (H+L chain) (Mouse) pAb-HRP	MBL	330	365	IB, 1:5000
Alexa Fluor TM 488 goat anti-mouse IgG (H+L)	Thermo Fisher Scientific (Waltham, MA, USA)	A11001	774-9040	IF, 1:500
Alexa Fluor TM 594 goat anti-mouse IgG (H+L)	Thermo Fisher Scientific	A11005	226-8383	IF, 1:500
Alexa Fluor TM 488 goat anti-rabbit IgG (H+L)	Thermo Fisher Scientific	A11008	075-1094	IF, 1:500
Alexa Fluor TM 594 goat anti-rabbit IgG (H+L)	Thermo Fisher Scientific	A11012	201-8240	IF, 1:500
Chemicals				
Curcumin	Santa Cruz Biotechnology	sc-200509	E3118	Final concentration, 10 microM
Dimethyl sulfoxide (DMSO)	FUJIFILM Wako Pure Chemical Corporation (Tokyo, Japan)	047-29353	CDN0170	Final concentration, less than 0.1%
Recombinant DNAs				
pCMV-SV40ori	A control constract deleted from Cat. No. 72035 (Addgene, Cambridge, MA, USA)	n.d.	n.d.	1.25 microgram of DNA per 6 cm dish
pCMV-SV40ori-mouse Sema5A-His	Addgene	72035	n.d.	1.25 microgram of DNA per 6 cm dish
pCMV-SV40ori-mouse Sema5A(R676C)-His	Generated in this study	n.d.	n.d.	1.25 microgram of DNA per 6 cm dish
pCMV-SV40ori-mouse Sema5A(R951C)-His	Generated in this study	n.d.	n.d.	1.25 microgram of DNA per 6 cm dish
pcDNA3.1-N-eGFP-RhoG-binding domain (RBD, amino acids 1-81) of human Elmo1 (synthesized in this study)	GenScript	J196YHL120-6	RG245690	1.25 microgram of DNA per 6 cm dish
pET42a-conserved Cdc42 and Rac interactive binding (CRIB) domain of human Pak1	Miyamoto, Y., Torii, T., Yamamori, N., Ogata, T., Tanoue, A., Yamauchi, J. Sci. Signal. 2013 6:ra15	n.d.	n.d.	2.5 microgram of DNA per 1 liter culture bottle for protein production

## Data Availability

The datasets used and/or analyzed for the current study are available from the corresponding author upon reasonable request.

## Supplementary Materials

The following supporting information can be downloaded at: https://www.mdpi.com/article/10.3390/pathophysiology30040040/s1, Figure S1. A neutralizing antibody for Plexin-B3, Plexin-A2, or Plexin-A3 inhibits morphological differentiation; Figure S2. JNK inhibitor recovers excessive process elongation induced by Sama5A harboring the R676C mutation; Figure S3. JNK inhibitor recovers excessive neuronal differentiation marker expression induced by Sama5A harboring the R676C mutation; Figure S4. RBD recovers excessive Rac1·GTP form induced by Sama5A harboring the R676C mutation; Figure S5. Original scan size blots for Figure 1; Figure S6. Original scan size blots for Figure 5; Figure S7. Original scan size blots for Figure 6; Figure S8. Original scan size blots for Figure 8; Figure S9. Original scan size blots for Figure 9; Figure S10. Original scan size blots for Figure S3; and Figure S11. Original scan size blots for Figure S4.
