# Supplementary material for: RhoG-Binding Domain of Elmo1 Ameliorates Excessive Process Elongation Induced by Autism Spectrum Disorder-Associated Sema5A
Source: Pathophysiology. 2023 Nov 27;30(4):548–66. doi: 10.3390/pathophysiology30040040 (PMC10745971; doi:10.3390/pathophysiology30040040)
Supplement: Supplementary file 1 [file pathophysiology-30-00040-s001.zip › 02.2. SEMA5A supple. leg.pdf]

Supplemental figures

Figure S1. A neutralizing antibody for Plexin-B3, Plexin-A2, or Plexin-A3 inhibits morphological differentiation. N1E-115 cells were allowed to differentiate in the presence or absence of control IgG (A-D) or a neutralizing antibody for Plexin-B3 (B), Plexin-A2 (C), or Plexin-A3 (D) for 48 hours. Differentiated cells were statistically depicted in the graph (** $p<0.01$; n = 10 fields).

Figure S2. JNK inhibitor recovers excessive process elongation induced by Sama5A harboring the R676C mutation. (A, B) Cells harboring Sema5A with the R676C mutation were treated in the presence or absence of a JNK inhibitor curcumin (10 microM) and allowed to differentiate for 0 or 48 hours. Differentiated cells were statistically depicted in the graph (** $p<0.01$; n = 10 fields). Some undissolved curcumin particles can be observed after the treatment with curcumin in (A).

Figure S3. JNK inhibitor recovers excessive neuronal differentiation marker expression induced by Sama5A harboring the R676C mutation. (A, B) The lysates of cells in the presence or absence of curcumin following the induction of differentiation were immunoblotted with an antibody against GAP43, Tau, or actin. Their immunoreactive band intensities were statistically depicted (* $p<0.05$, ** $p<0.01$; n = 3 blots). An anti-Tau antibody recognizes approximately 50 kDa of many Tau protein variants. Other immunoreactive bands can be non-specific ones.

Figure S4. RBD recovers excessive Rac1·GTP form induced by Sama5A harboring the R676C mutation. (A, B) The lysates of cells following the induction of differentiation were affinity-precipitated with the recombinant GST-CRIB and immunoblotted with an anti-Rac1 antibody. The lysates were immunoblotted with an antibody against Rac1, His-tag, GFP-tag, or actin to monitor total proteins used in affinity-precipitation. Their immunoreactive band intensities were statistically depicted (** $p<0.01$; n = 3 blots).

Figure S5. Original scan size blots for Figure 1.

Figure S6. Original scan size blots for Figure 5.

Figure S7. Original scan size blots for Figure 6.

Figure S8. Original scan size blots for Figure 8.

Figure S9. Original scan size blots for Figure 9.

Figure S10. Original scan size blots for Figure S3.

Figure S11. Original scan size blots for Figure S4.
