# Supplementary figures and images for: RhoG-Binding Domain of Elmo1 Ameliorates Excessive Process Elongation Induced by Autism Spectrum Disorder-Associated Sema5A
Source: Pathophysiology. 2023 Nov 27;30(4):548–66. doi: 10.3390/pathophysiology30040040 (PMC10745971; doi:10.3390/pathophysiology30040040)

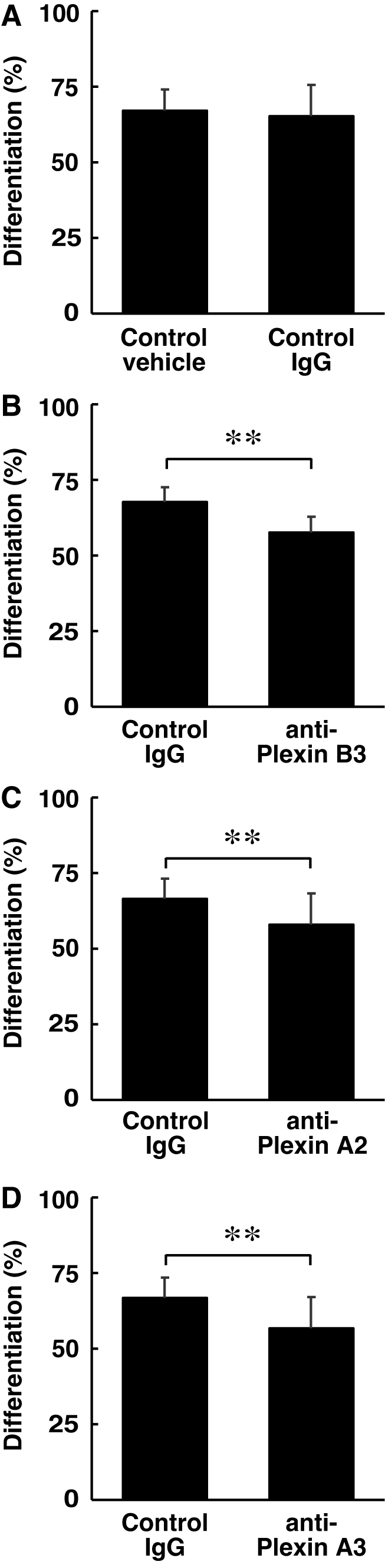

Supplement: Supplementary file 1 [file pathophysiology-30-00040-s001.zip › Figure S1.tif]

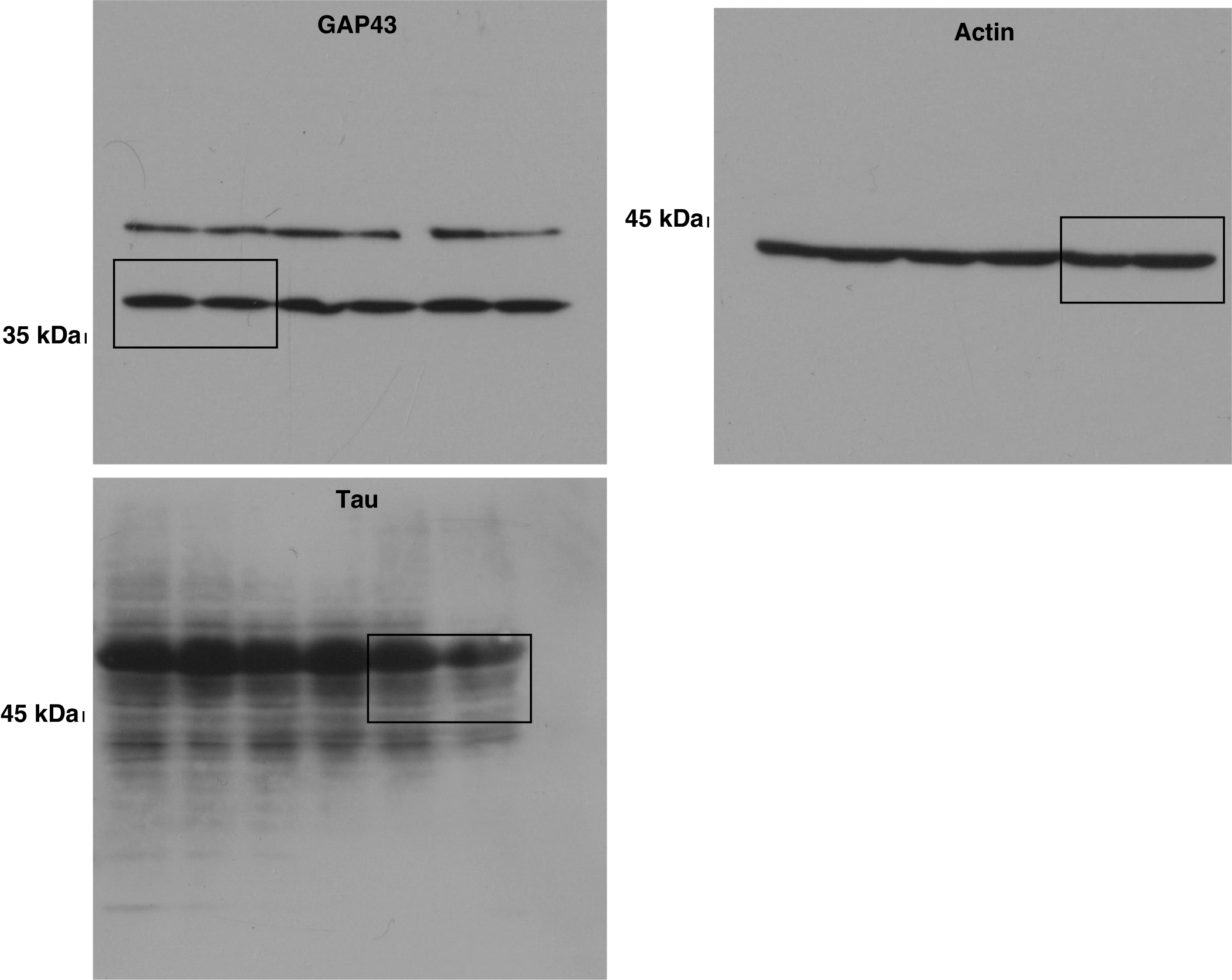

Supplement: Supplementary file 1 [file pathophysiology-30-00040-s001.zip › Figure S10 Supplemental file for Figure S3.tif]

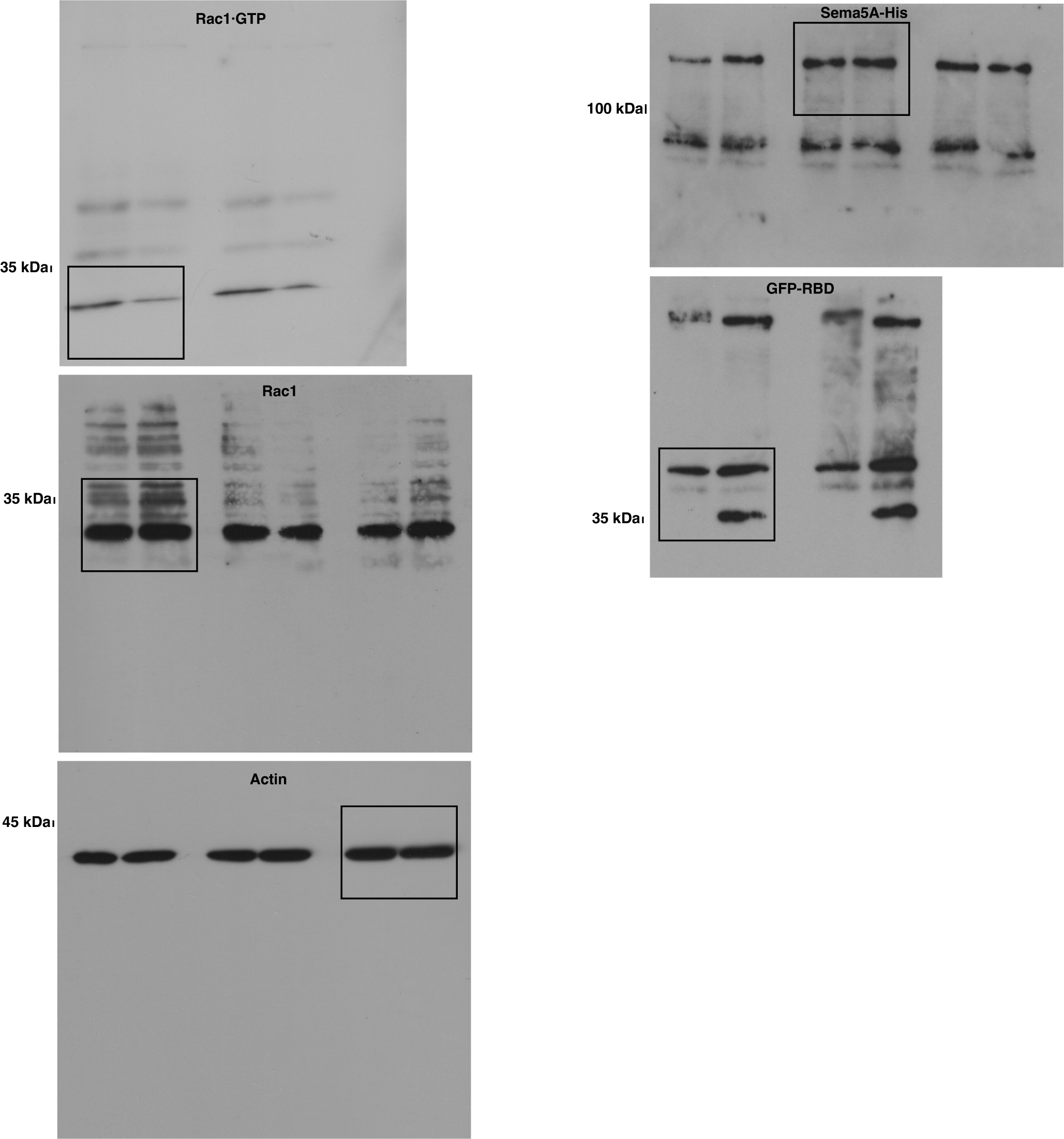

Supplement: Supplementary file 1 [file pathophysiology-30-00040-s001.zip › Figure S11 Supplemental file for Figure S4.tif]

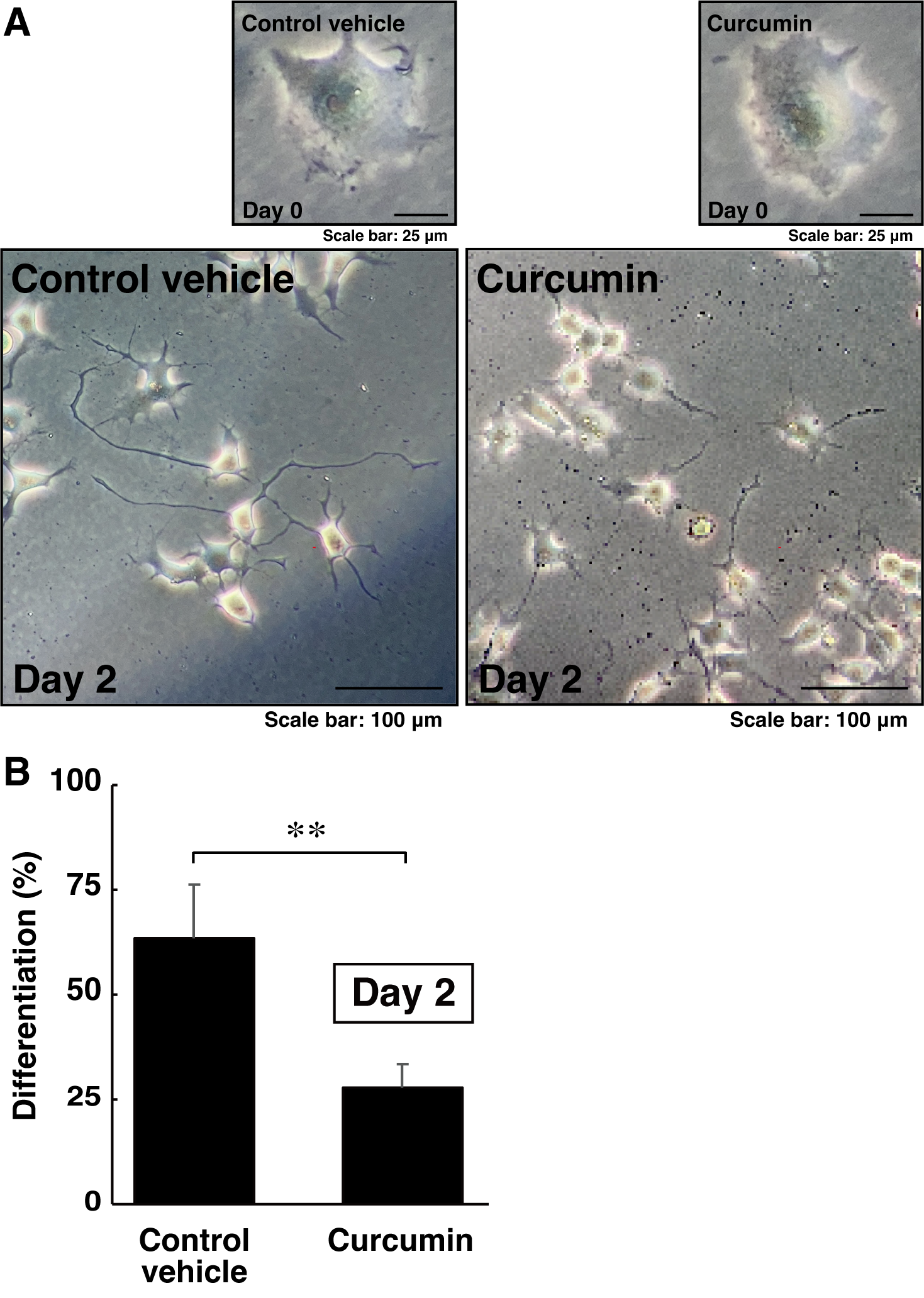

Supplement: Supplementary file 1 [file pathophysiology-30-00040-s001.zip › Figure S2.tif]

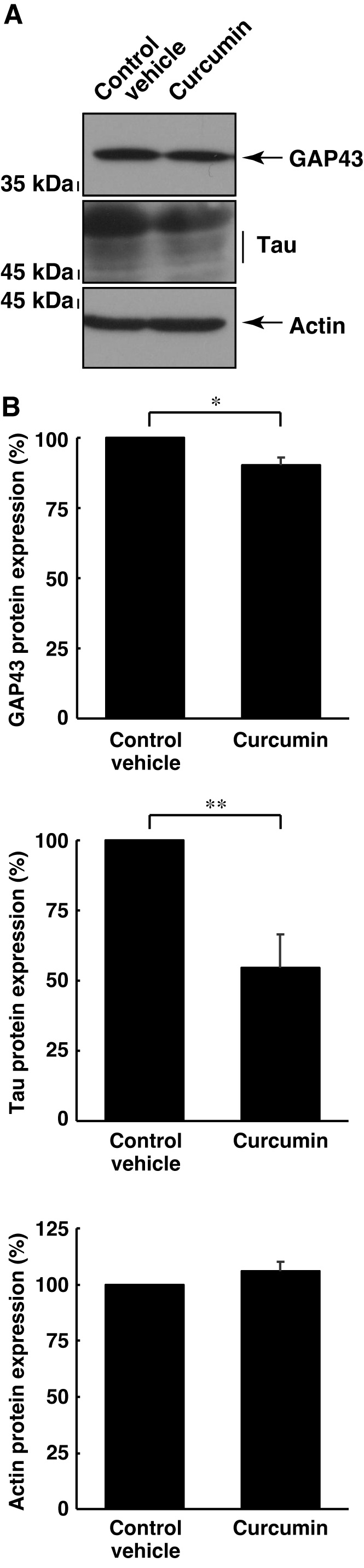

Supplement: Supplementary file 1 [file pathophysiology-30-00040-s001.zip › Figure S3.tif]

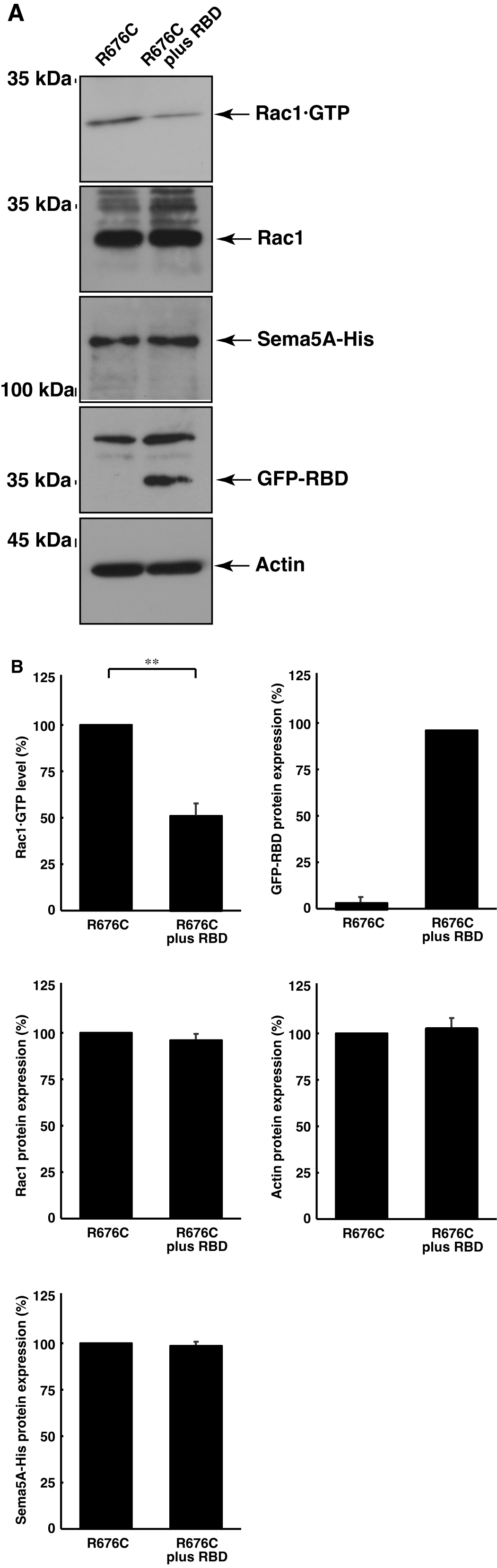

Supplement: Supplementary file 1 [file pathophysiology-30-00040-s001.zip › Figure S4.tif]

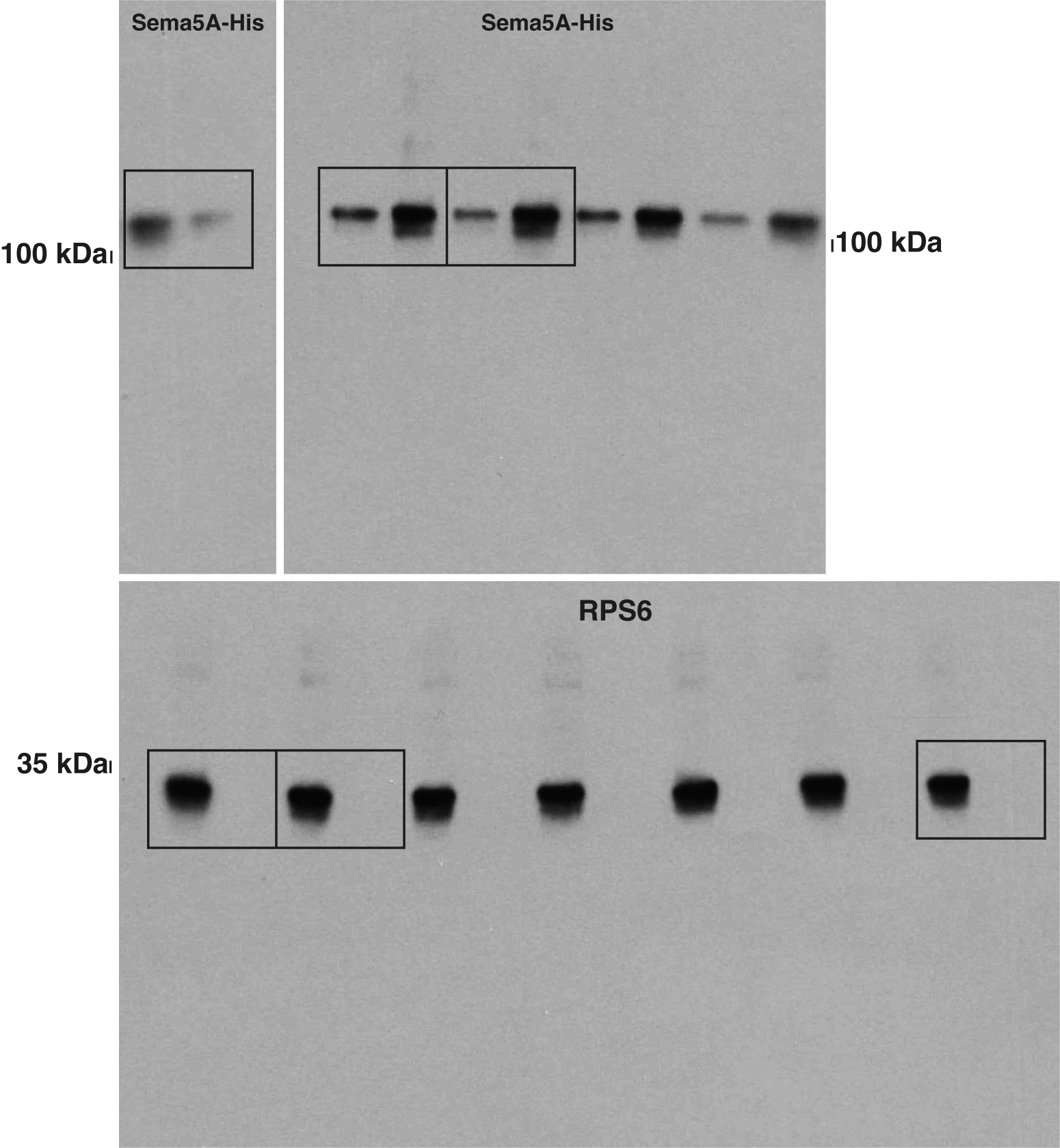

Supplement: Supplementary file 1 [file pathophysiology-30-00040-s001.zip › Figure S5 Supplemental file for Figure 1.tif]

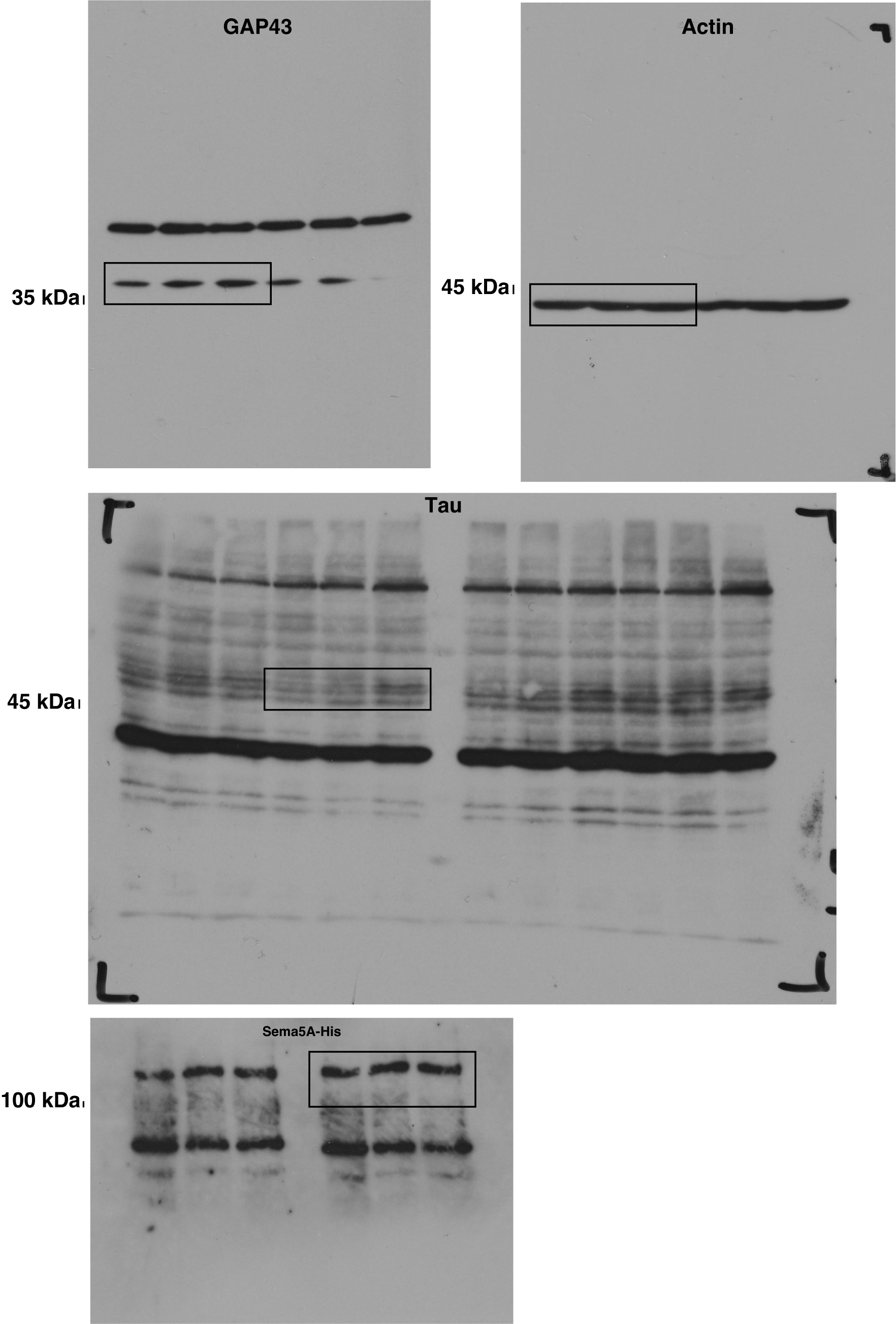

Supplement: Supplementary file 1 [file pathophysiology-30-00040-s001.zip › Figure S6 Supplemental file for Figure 5.tif]

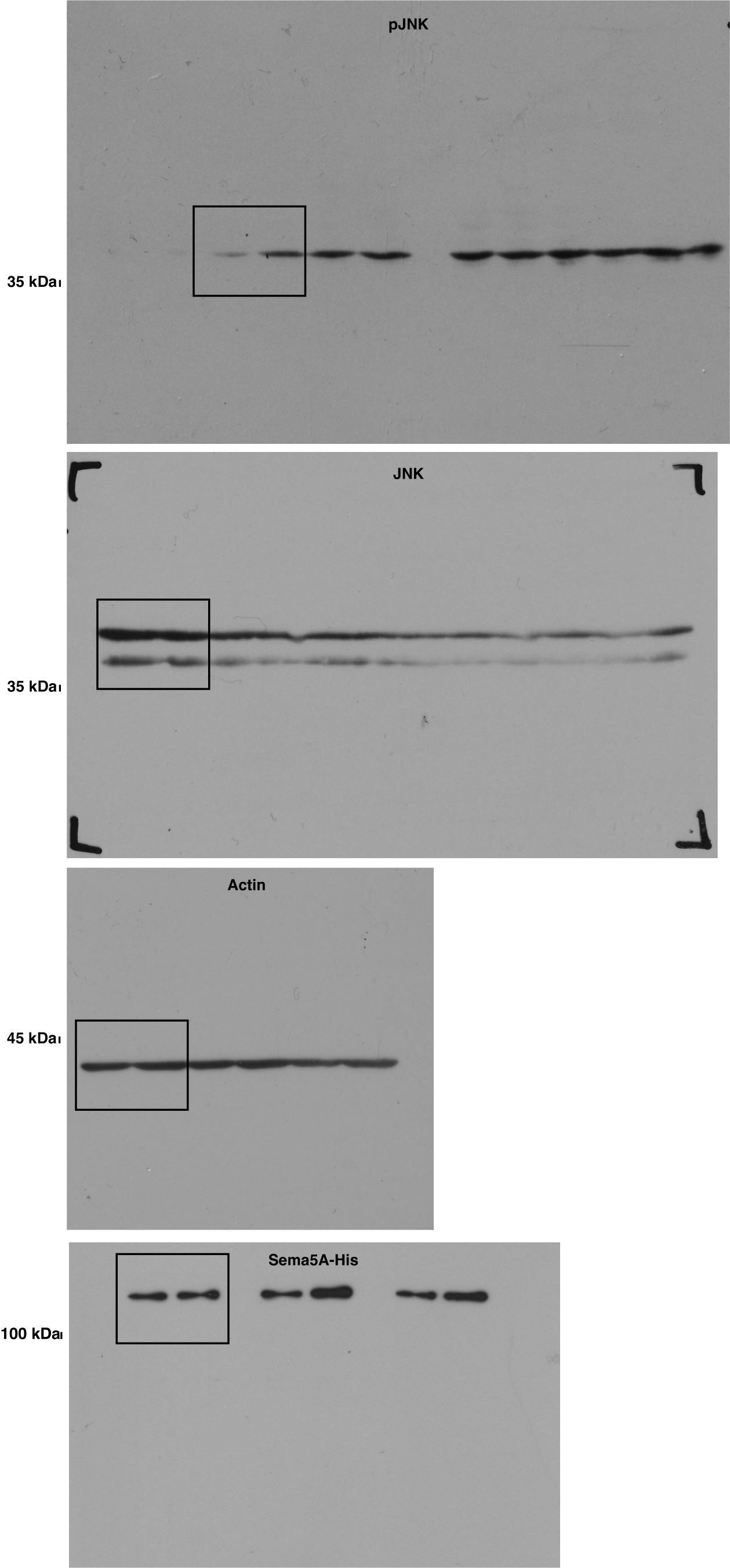

Supplement: Supplementary file 1 [file pathophysiology-30-00040-s001.zip › Figure S7 Supplemental file for Figure 6.tif]

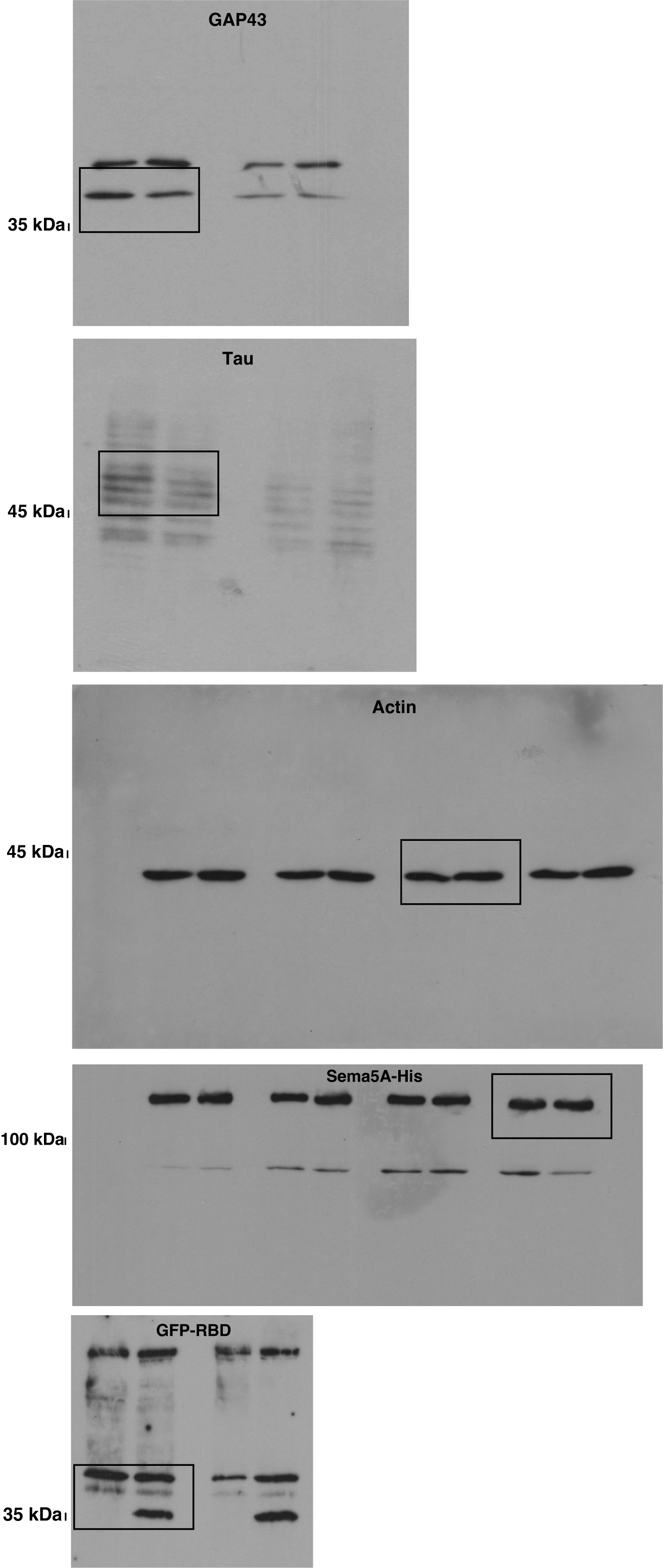

Supplement: Supplementary file 1 [file pathophysiology-30-00040-s001.zip › Figure S8 Supplemental file for Figure 8.tif]

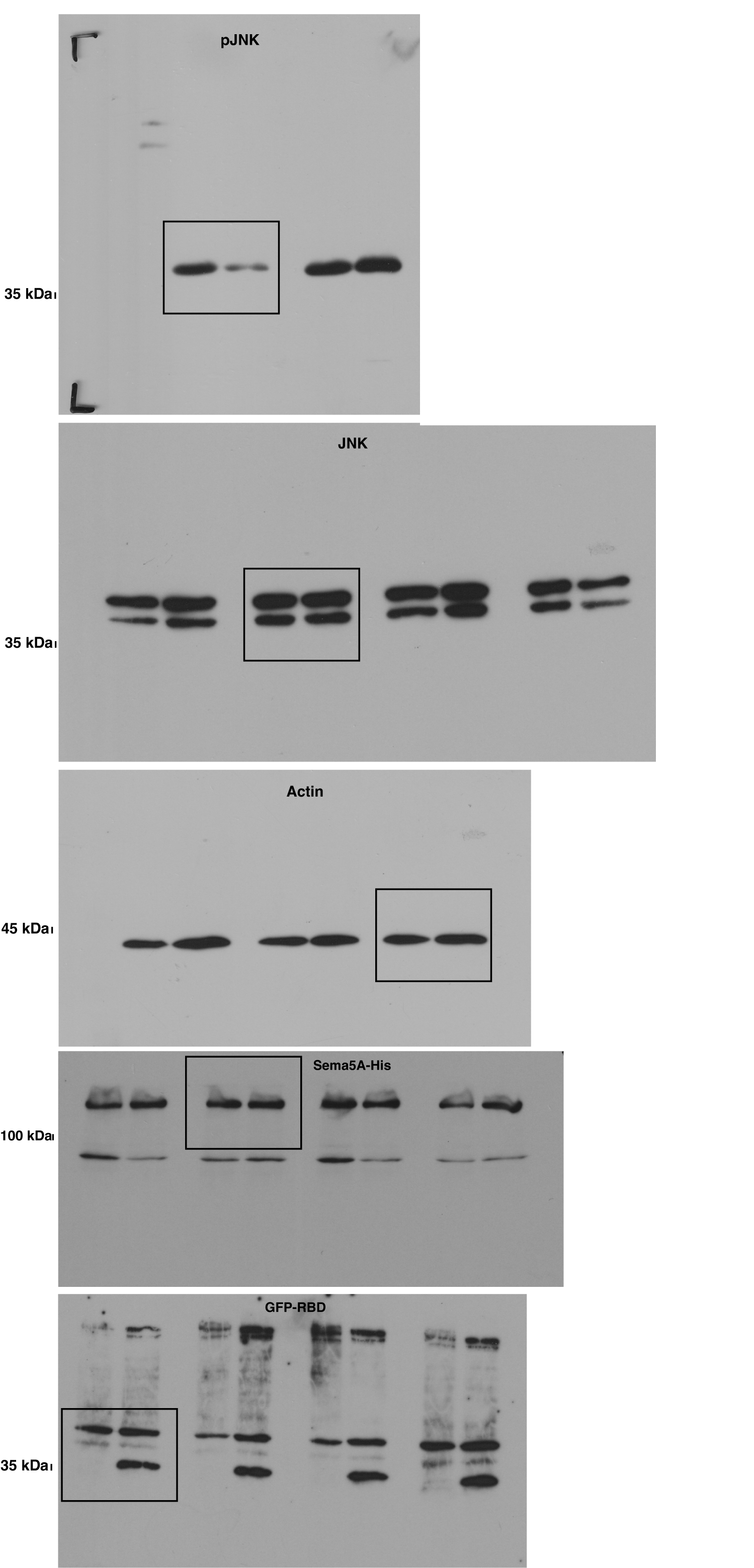

Supplement: Supplementary file 1 [file pathophysiology-30-00040-s001.zip › Figure S9 Supplemental file for Figure 9.tif]
